# Patients’ Opinions towards the Services of Pharmacists Based in General Practice

**DOI:** 10.3390/pharmacy10040078

**Published:** 2022-07-07

**Authors:** Thilini Sudeshika, Mark Naunton, Kwang C. Yee, Louise S. Deeks, Gregory M. Peterson, Sam Kosari

**Affiliations:** 1 Discipline of Pharmacy, Faculty of Health, University of Canberra, Bruce, ACT 2617, Australia; mark.naunton@canberra.edu.au (M.N.); kwang_choon.yee@canberra.edu.au (K.C.Y.); louise.deeks@canberra.edu.au (L.S.D.); g.peterson@utas.edu.au (G.M.P.); sam.kosari@canberra.edu.au (S.K.); 2 Department of Pharmacy, Faculty of Allied Health Sciences, University of Peradeniya, Peradeniya 20400, Sri Lanka; 3 School of Pharmacy and Pharmacology, University of Tasmania, Hobart, TAS 7005, Australia

**Keywords:** patient opinions, awareness, perceived needs, satisfaction, general practice pharmacist

## Abstract

Pharmacists have been included in general practice teams to provide non-dispensing services for patients. In Australia, pharmacists’ role in general practice has been slowly expanding. However, there is a paucity of research to explore patients’ opinions toward pharmacist-led services in general practice. This study aimed to assess patient awareness, perceived needs, and satisfaction with these services. A cross-sectional survey was conducted with a purposeful sample of patients who visited six general practices in the Australian Capital Territory that included pharmacists in their team. The survey was informed by the literature and pre-tested. The survey was distributed to two samples: patients who had seen a pharmacist and those who had not seen a pharmacist. Of 100 responses received, 86 responses were included in the analysis: patients who had seen a pharmacist (*n* = 46) and patients who had not seen a pharmacist (*n* = 40). Almost all the patients who utilised pharmacist-led services were highly satisfied with those services. Among patients who had not seen a pharmacist, 50% were aware of the existence of general practice pharmacists. Patients who had visited the pharmacist rated higher scores for perceived needs. Patient satisfaction towards the pharmacist-led services in general practices was very high, and patients supported the expansion of these services. However, awareness of the availability of general practice pharmacist services could be improved.

## 1. Introduction

As team-based care is evolving worldwide, pharmacists have been included in general medical practices to provide non-dispensing services for patients [1,2]. The primary purpose of including pharmacists in general practice teams is to optimise medication use and minimise medicine-related harm [2,3,4]. Pharmacist-led services in general practices include medication management services, medication safety initiatives, and providing education [5]. The inclusion of pharmacists in general practice teams has been studied in a number of countries, with results showing that pharmacists can provide a range of services to benefit patients [6,7,8,9,10,11].

In Australia, previous studies have indicated that the role of pharmacists in general practices was well accepted by stakeholders, including general practitioners (GPs), practice managers and consumers, at the initiation of general practice pharmacists’ roles [12,13,14,15,16]. However, studies related to patient perspectives following the inclusion of pharmacists in general practice teams are sparse [17]. Moreover, a limited number of pharmacists have been employed in general practices across Australia, so pharmacist-led services in general practices are relatively new to patients [17,18]. Therefore, there is a need to gain a better understanding of patients’ awareness, utilisation, and perceptions of pharmacist-led services in this setting.

In the Australian Capital Territory (ACT), general practice pharmacist-led services were first established in 2016 through funding from the Capital Health Network (CHN: Primary Health Network in the ACT) [19,20]. This cross-sectional study is part of a broader study to evaluate the inclusion of pharmacists in general practice, which is a relatively new initiative in Australia. Thus, the objective of this study was to assess the opinions of patients on their awareness, perceived needs, and satisfaction with the services of these pharmacists.

## 2. Materials and Methods

### 2.1. Ethics Approval

This study was approved by the Human Research Ethics Committee of the University of Canberra (HREC 15-235) on 2 December 2019.

### 2.2. Intervention

Pharmacists were initially employed in eight general practices in the ACT on a part-time basis (15 h per week) for 18 months to deliver collaborative care with other general practice team members. Pharmacists provided non-dispensing services, such as medication reviews/medication management services, patient education, medication safety initiatives, and medication information to general practice staff [21]. These pharmacists were funded by the CHN, and pharmacists’ services for patients were free of charge.

### 2.3. Design and Setting

As part of a broader study to evaluate the inclusion of pharmacists in general practice in the ACT, a survey was utilised to explore patient opinions on general practice pharmacists’ services [22,23,24]. The eight general practices that included pharmacists in their teams were invited to participate in this cross-sectional survey-based study. Six practices agreed to participate. Of the two practices that declined to participate, one declined due to a shortage of staff and the other because the pharmacist left general practice within 10 months.

The survey (Additional File S1) was developed by considering prior studies [12,13,14,25,26]. It comprised three main domains: demographic details; awareness of general practice pharmacists amongst patients and perceived needs towards the pharmacist-led services; and satisfaction with the services. Patients’ age, gender, level of education, reason for visiting the general practice, length of using the general practice, and type of health insurance were requested in demographic details. Patients rated their perceived needs and satisfaction via a numerical rating scale. In the domain ‘perceived needs’, respondents were asked to rate the importance of six primary activities that pharmacists could perform in general practices, on a 0 to 10-point numerical scale ranging from “0 = not important” to “10 = extremely important”. Those activities were “Improving the safety of medicines”, “Improving the effectiveness of medicines”, “Assessing potential or actual adverse effects of medicines”, “Developing a plan with doctors to manage a medical condition”, “Providing education about medicines”, and “Providing education to modify lifestyle”. Statements for satisfaction were adapted from a previously validated tool [26]. This domain contained four sub-domains to assess the satisfaction of professional care provided by pharmacists, the relationship between the pharmacist and patient, the length of the consultation, and general satisfaction. For these questions, respondents were asked to rate their agreement on a 0 to 10-point numerical scale ranging from “0 = strongly disagree” to “10 = strongly agree”. At the end of the survey, respondents were asked to indicate how much they would be willing to pay if they had to pay for a 40-min consultation with a general practice pharmacist. The time of the consultation with the general practice pharmacist was decided by considering the results of a pilot study [14]. The survey underwent face validation (five experts who were pharmacists and academics and had practice-based experience of more than 10 years each) and was pre-tested before distribution (two patients).

### 2.4. Participants, Recruitment, and Data Collection

Two samples of patients were recruited from the study sites to represent patients who had seen and not seen the general practice pharmacists between 2020 and 2021. Patients in both groups were asked to report their awareness and perceived needs for general practice pharmacist services. Only patients who had seen the general practice pharmacists reported their satisfaction with pharmacist-led services and willingness to pay. Surveys were provided to the general practices (50 per practice) at 10 months following the inclusion of pharmacists in general practice teams. To prevent the risk of multiple submissions from the same respondent, pharmacists distributed surveys to patients who had visited the pharmacist, and receptionists distributed surveys to patients who had not visited the pharmacist. Responses were collected while maintaining respondent confidentiality using sealed envelopes and locked boxes. The questionnaires were available to patients in general practices for 4–6 months. Posters were displayed in the general practices to increase the response rate.

### 2.5. Data Analysis

Descriptive statistics (frequencies, means and standard deviations (SD) or median and ranges) were used to summarise the data. Mann–Whitney U tests were performed to compare the scores for perceived needs between the patients who had seen or not seen the general practice pharmacists. A *p*-value < 0.05 was considered statistically significant. The data were analysed by using Statistical Package for the Social Sciences (SPSS ver. 27 IBM, New York, NY, USA).

## 3. Results

### 3.1. Demographics

A total of 230 from the 300 available questionnaires were distributed to patients or carers by pharmacists or receptionists across six general practices, and 100 questionnaires were returned. Fourteen questionnaires with predominantly incomplete or ambiguous responses were excluded. Of 86 responses, 46 responses were from patients who had visited the general practice pharmacist and 40 responses were from patients who had not visited the pharmacist (Table 1). As anticipated, the pharmacists were more likely to see older patients with chronic medical conditions.

### 3.2. Awareness and Perceived Needs

Among the patients who had not seen the pharmacist in general practice, 50% of patients (*n* = 20) were aware of pharmacist-led services available in their general practice. Respondents who had seen the general practice pharmacist rated higher scores for the perceived need for pharmacist-led services (Table 2).

### 3.3. Satisfaction towards the Pharmacist-Led Services

Among the respondents who had utilised general practice pharmacist-led services, 43% (*n* = 20) had visited the pharmacist for the first time. The most common reasons for visiting the pharmacist were medication management services/reviews (*n* = 18, 39%); as part of health assessments (government-funded) [27] (*n* = 9, 20%); vaccinations (*n* = 9, 20%); and other reasons, such as asthma care, diabetes education, and smoking cessation (*n* = 8, 17%). Most patients (*n* = 31, 67%) had been referred to the pharmacist by a GP (Table S1).

Satisfaction toward the services of general practice pharmacists was high across a range of measures (Table 3). If the patients had to pay for a 40-min consultation with a general practice pharmacist, 61% of patients (*n* = 28) reported that they would be willing to pay a mean amount of AUD 58.2 ± 37.7, while 11% (*n* = 5) reported an unwillingness to pay and 28% (*n* = 13) did not answer the question.

## 4. Discussion

Pharmacist services in general practice are relatively new in Australia. Therefore, the perception of patients is a key consideration to successfully introducing pharmacists to the general practice setting on a wider scale. While prior studies reported only patient satisfaction towards the services of pharmacists in general practice [14,17,25], this is the first study in Australia reporting patient awareness and perceived needs towards the general practice pharmacist-led services after including pharmacists in general practice teams. Our findings suggest that pharmacists’ services were professional and patient satisfaction with pharmacists’ services was high. However, it seems essential to improve patient awareness of pharmacists’ services in general practice. Understanding patient awareness, perceived needs, and satisfaction towards the services of general practice pharmacists are necessary to successfully implement and utilise these new non-dispensing services of pharmacists. Therefore, our findings may be beneficial to stakeholders, pharmacists, researchers and policymakers to guide interventions in Australia and in other countries that wish to introduce non-dispensing pharmacists into general practices.

Our findings indicated that among the patients who had not seen a general practice pharmacist, 50% of patients were aware of the existence of pharmacists in general practice. This finding is consistent with qualitative studies conducted with patients in the UK [28,29]. Furthermore, and perhaps not surprisingly, patients who had seen the pharmacist rated higher scores for all the perceived needs for the services of general practice pharmacists. Patients who had seen the general practice pharmacist seemed to have a very high appreciation of the services. It has been reported that patients’ awareness and expectations can significantly influence patients’ health outcomes [30]. Understanding and managing patients’ awareness and expectations may also lead to increased satisfaction with healthcare services [30,31].

Patients who utilised the pharmacist services were highly satisfied with the care provided by the pharmacists, the length of the pharmacist–patient consultation and their relationship with the pharmacist. These findings are consistent with prior studies in Australia and elsewhere [14,25,32,33,34]. Our findings showed that most patients visited the general practice pharmacist for medication management services/reviews, vaccinations, and health assessments. Medication management services were the primary role of the general practice pharmacists, as identified in other studies [17,35,36]. Respondents strongly agreed that they would visit the general practice pharmacist in the future; this was similar to the finding in another Australian study [25]. Patient satisfaction is one of the most common performance indicators of healthcare quality [37]. It can affect the loyalty of patients, patient retention for services, and productivity of staff [37,38]. The high patient satisfaction in this study supports expanding the introduction of pharmacists in the Australian general practice setting.

There are limitations to this study. Even though the response rate to the questionnaire was acceptable, responses were limited to six general practices in the ACT, suggesting the findings may not be generalisable [39]. Furthermore, it is not possible to conclude that non-respondents would have held similar opinions on awareness, perceived needs, and satisfaction with general practice pharmacists’ services. The questionnaires were also distributed to patients by reception staff and general practice pharmacists, which may have introduced selection bias. Thus, further research is recommended with larger random samples. There is a possibility of response bias and confirmation bias in this study. However, strategies, such as utilising a numerical scale, recruiting patients who had seen/not seen the pharmacist, ensuring anonymity, and adapting statements from a validated tool were considered in designing this study to minimise the bias [40]. Surveys are useful tools to assess patient opinions; however, considering the potential limitations of a survey-based study to assess patient perspectives, a qualitative study is recommended to gain insight into patient perspectives towards the services of general practice pharmacists in Australia.

## 5. Conclusions

This study has revealed that patients’ satisfaction with pharmacist-led services in general practices was high. Patients found that the services were convenient, useful, and professional. Furthermore, patients showed support and interest to expand the services of pharmacists in the Australian general practice setting. Awareness of the availability of general practice pharmacists’ services could be improved.

## Figures and Tables

**Table 1:** Demographics of the respondents.

Variable	Patients Who Had Seen the General Practice Pharmacist *n* = 46	Patients Who Had Not Seen the General Practice Pharmacist *n* = 40
Age (mean ± SD) years	51.6 ± 24.4	35.6 ± 24.2
Gender (*n*, %)		
Male	22 (48)	23 (58)
Female	19 (41)	16 (40)
Other/prefer not to say	3 (7)	0
Not answered	2 (4)	1 (3)
Education (*n*, %)		
No formal education	4 (9)	4 (10)
Up to Year 6 or equivalent	5 (11)	1 (3)
Up to Year 12 or equivalent	8 (17)	14 (35)
Vocational training	13 (28)	7 (18)
Bachelor’s degree	8 (17)	2 (5)
Postgraduate degree	4 (9)	6 (15)
Other	4 (9)	6 (15)
Reason for visiting the general practice (*n*, %)		
Because of one-off problem/condition/illness	1 (2)	15 (38)
Because of a long-term problem/condition/illness, where treatment has been started/changed within the last 12 months	18 (39)	9 (23)
Because of a long-term problem/condition/illness, where treatment has not been started/changed within the last 12 months	10 (22)	5 (13)
For a health check but has not been diagnosed with any disease previously	3 (7)	6 (15)
Pregnancy	1 (2)	2 (5)
Other	12 (26)	3 (8)
Not answered	1 (2)	0
Length of using the general practice (*n*, %)		
This is the first time	3 (7)	11 (28)
Up to 6 months	11 (24)	8 (20)
Up to 12 months	5 (11)	8 (20)
More than 12 months	27 (59)	13 (33)
Type of health insurance (*n*, %)		
Medicare (government-funded healthcare insurance)	22 (48)	16 (40)
Private	3 (7)	4 (10)
Medicare and private	17 (37)	16 (40)
None	4 (9)	3 (8)
Not answered	0	1 (3)

**Table 2:** Patient-rated scores for perceived needs towards the services of general practice pharmacists.

Perceived Needs	Patients Who Had Seen the Pharmacist *n* = 46 Median (Range)	Patients Who Had Not Seen the Pharmacist *n* = 40 Median (Range)	Mann-Whitney U Test *p*-Value
Improving the safety of medicines	9 (4–10)	8 (5–10)	0.005
Improving the effectiveness of medicines	9 (4–10)	8 (5–10)	0.013
Assessing potential or actual adverse effects of medicines	9 (4–10)	8 (5–10)	0.002
Developing a plan with doctors to manage a medical condition	9 (4–10)	7 (3–10)	0.002
Providing education about medicines	9 (4–10)	8 (3–10)	0.002
Providing education to modify lifestyle	8 (2–10)	5 (0–10)	0.002

**Table 3:** Patient-rated satisfaction towards the services of general practice pharmacists.

Item/Statement	Median (Range)
This general practice pharmacist was very careful to check all medicines that I was taking	9 (7–10)
This general practice pharmacist conducted the session with respect to me as a person	10 (8–10)
This general practice pharmacist explained the treatment in a way that I can understand	10 (7–10)
I will follow the advice of this pharmacist because I think he/she is right	10 (8–10)
The time of the consultation with the pharmacist was enough to discuss everything	9 (4–10)
I wish it had been possible to spend a little longer with the pharmacist	7 (0–10)
The time I was able to spend with the pharmacist was a bit too short	4 (0–10)
There are some facts that this general practice pharmacist did not seem to understand about me	2 (0–10)
I felt able to tell this general practice pharmacist about personal facts	9 (7–10)
I felt this general practice pharmacist really knew what I was thinking	8 (0–10)
I have a good relationship with this general practice pharmacist	9 (6–10)
The time I spent with this general practice pharmacist was very productive	9 (5–10)
The consultation with this general practice pharmacist could have been better	1 (0–10)
I would visit this general practice pharmacist in the future	10 (5–10)
I would recommend the service of a general practice pharmacist to other patients	10 (0–10)
I was completely satisfied with the visit to the general practice pharmacist	10 (5–10)

## Data Availability

Not applicable.

## Supplementary Materials

The following supporting information can be downloaded at: https://www.mdpi.com/article/10.3390/pharmacy10040078/s1, Additional File S1: Patients’ Opinions Survey; Table S1: Details of patient visits to general practice pharmacist.
