# Supplementary material for: Patients’ Opinions towards the Services of Pharmacists Based in General Practice
Source: Pharmacy (Basel). 2022 Jul 7;10(4):78. doi: 10.3390/pharmacy10040078 (PMC9326583; doi:10.3390/pharmacy10040078)
Supplement: Supplementary file 1 [file pharmacy-10-00078-s001.zip › Additional File S1.pdf]

Additional File S1: Patients' Opinions Survey

Patients' Opinions Survey

Are you filling in this questionnaire as a: ☐ Patient ☐ Carer ☐ Parent/guardian

If you are filling in this survey as a carer/ parent, it is important that the patient's opinions are presented in questions in part 2 and 3.

Please note: In this survey, the word "*on-site pharmacist***" is used for describing pharmacist that working at the GP-clinic, which is different from pharmacist at your local community pharmacy (also known as a local chemist)

PART 1 – Basic information

(1) **How old is the patient?** ____________ years

(2) **Is the patient?** ☐ Male ☐ Female ☐ Other / Prefer not to say

(3) **What is patient's highest level of education?**

☐ No formal education ☐ Up to Year 6 or equivalent

☐ Up to Year 12 or equivalent ☐ Vocational training (e/g. TAFE, Diploma)

☐ Bachelor degree ☐ Postgraduate degree (e/g. Masters, PhD)

☐ Other (please specify) ____

(4) **How long has the patient been visiting this general practice?**

☐ This is the first time ☐ Up to 6 months

☐ Up to 12 months ☐ More than 12 months

(5) **Which of the following best describes the patient's reason for visiting this general practice today?**

☐ Because of a one-off problem/ condition/ illness (e.g. flu, injury)

☐ Because of a long-term problem/ condition/ illness (e.g. diabetes, heart disease), where treatment has been started/ changed within the last 12 months

☐ Because of a long-term problem/ condition/ illness (e.g. diabetes, heart disease), where treatment has not been changed within the last 12 months

☐ For a health check but have not been diagnosed with any disease previously

☐ Pregnancy/ family planning

☐ Other (please specify)

____

(6) **What type of health insurance does patient have?**

☐ Medicare ☐ Private ☐ Both ☐ None

PART 2 – Awareness and Attitudes

(1) **This practice has employed an *on-site pharmacist* since the past year. Which of the following best describes your awareness towards the on-site pharmacist?**

☐ I've never heard about the pharmacist in this general practice

☐ I've heard about the pharmacist in this general practice, but I've not visited him/ her

☐ I've heard about the pharmacist in this general practice, and I've visited him/her

(2) **If you have visited the *on-site pharmacist*, who referred you (or the patient that you care for) to the pharmacist?**

☐ Doctor ☐ Nurse ☐ Receptionist ☐ Psychologist

☐ Self ☐ Other (please specify)___________________________________

(3) **How do you rate the importance of the following activities that could be performed by the *on-site pharmacist*?**

Circle the number that best describes your opinion. (0 = Not important, 10 = Extremely important, N/ A = Not applicable)

Improving the safety of medicines

0 1 2 3 4 5 6 7 8 9 10 No opinion or N/A

Improving the effectiveness of medicines

0 1 2 3 4 5 6 7 8 9 10 No opinion or N/A

Assessing potential or actual adverse effects of medicines

0 1 2 3 4 5 6 7 8 9 10 No opinion or N/A

Developing a plan with doctors to manage a medical condition

0 1 2 3 4 5 6 7 8 9 10 No opinion or N/A

Providing education on medicines

0 1 2 3 4 5 6 7 8 9 10 No opinion or N/A

Providing education to modify lifestyle

0 1 2 3 4 5 6 7 8 9 10 No opinion or N/A

If you have visited the pharmacist in this general practice, please continue to part 3 on next page

If you have not visited a pharmacist in this general practice, the survey ends here.

PART 3 – Satisfaction

(1) **Which of the following best describes the reason that you (or the patient that you care for) were referred to the *on-site pharmacist*? *(Tick all the options that apply for you)***

☐ For a routine check to verify my medicines are working for me

☐ To ask for advice to modify lifestyle ☐ To check for drug interactions

☐ To report adverse effects ☐ For a health assessment

☐ For vaccination

☐ Other (please specify) ____

(2) **How many times have you (or the patient that you care for) seen the *on-site pharmacist* in total?**

☐ 1 ☐ 2 ☐ 3 ☐ 4 ☐ 5 or more times

(3) **At the most recent visit to the *on-site pharmacist*, how would you rate <u>your experience</u> of the following?**

<u>Circle the number</u> that best describes your opinion. (0 = Strongly disagree, 10 = Strongly agree, N/ A = Not applicable)

This *on-site pharmacist* was very careful to check all medicines that I (or the patient that I care for) was taking

0 1 2 3 4 5 6 7 8 9 10 No opinion or N/A

This *on-site pharmacist* conducted the session with respect to me as a person

0 1 2 3 4 5 6 7 8 9 10 No opinion or N/A

This *on-site pharmacist* explained the treatment in a way that I can understand

0 1 2 3 4 5 6 7 8 9 10 No opinion or N/A

I will follow the advice of this *on-site pharmacist* because I think he/she is right

0 1 2 3 4 5 6 7 8 9 10 No opinion or N/A

(4) **How would you rate the <u>timing of your consultation</u> with the *on-site pharmacist*?**

<u>Circle the number</u> that best describes your opinion. (0 = Strongly disagree, 10 = Strongly agree, N/ A = Not applicable)

The time of the consultation with the pharmacist was enough to discuss everything

0 1 2 3 4 5 6 7 8 9 10 No opinion or N/A

I (or the patient that I care for) wish it had been possible to spend a little longer with the pharmacist

0 1 2 3 4 5 6 7 8 9 10 No opinion or N/A

The time I (or the patient that I care for) was able to spend with the pharmacist was a bit too short

0 1 2 3 4 5 6 7 8 9 10 No opinion or N/A

(5) **How would you rate <u>your relationship</u> towards the *on-site pharmacist*?**

<u>Circle the number</u> that best describes your opinion. (0 = Strongly disagree, 10 = Strongly agree, N/ A = Not applicable)

There are some facts that this *on-site pharmacist* did not seem to understand about me (or the patient that I care for)

0 1 2 3 4 5 6 7 8 9 10 No opinion or N/A

I (or the patient that I care for) felt able to tell this *on-site pharmacist* about personal facts

0 1 2 3 4 5 6 7 8 9 10 No opinion or N/A

I felt this *on-site pharmacist* really knew what I (or the patient who I care for) was thinking

0 1 2 3 4 5 6 7 8 9 10 No opinion or N/A

I (or the patient that I care for) have a good relationship with this *on-site pharmacist*

0 1 2 3 4 5 6 7 8 9 10 No opinion or N/A

(6) How would you rate <u>your satisfaction</u> towards the on-site pharmacist?

<u>Circle the number</u> that best describes your opinion. (0 = Strongly disagree, 10 = Strongly agree, N/ A = Not applicable)

The time I (or the patient that I care for) spent with this *on-site pharmacist* was very productive

0 1 2 3 4 5 6 7 8 9 10 No opinion or N/A

The consultation with this *on-site pharmacist* could have been better

0 1 2 3 4 5 6 7 8 9 10 No opinion or N/A

I (or the patient that I care for) would visit this *on-site pharmacist* in the future

0 1 2 3 4 5 6 7 8 9 10 No opinion or N/A

I would recommend the service of an *on-site pharmacist* in general practice to other patients

0 1 2 3 4 5 6 7 8 9 10 No opinion or N/A

I (or the patient that I care for) was completely satisfied with the visit to the *on-site pharmacist* in this general practice

0 1 2 3 4 5 6 7 8 9 10 No opinion or N/A

(7) **If the patient had to pay for a 40-minute consultation with an *on-site pharmacist*, how much would you be willing to pay?** AUD______________________

Thank you for your time and participation. Now please put the survey in the envelope provided and place it in the black box available at the reception desk.
