# Supplementary material for: Patients’ Opinions towards the Services of Pharmacists Based in General Practice
Source: Pharmacy (Basel). 2022 Jul 7;10(4):78. doi: 10.3390/pharmacy10040078 (PMC9326583; doi:10.3390/pharmacy10040078)
Supplement: Supplementary file 1 [file pharmacy-10-00078-s001.zip › Table S1.pdf]

Table S1: Details of patient visits to general practice pharmacists

Detail	Respondents n=46 (%)
Frequency of visiting the pharmacist	
First time	20 (44)
2 times	8 (17)
3 times	9 (20)
4 times	2 (4)
5 times or more	6 (13)
Not answered	1 (2)
Reason for visiting the pharmacist (allowed to select all the applicable options)	
For a routine check to verify my medicines are working for me	18 (39)
To ask for advice to modify lifestyle	5 (11)
To check for drug interactions	3 (7)
To report adverse effects	4 (9)
For a health assessment	9 (20)
For vaccination	9 (20)
Other	8 (17)
Referral to the pharmacist by	
General practitioner	31 (67)
Nurse	4 (9)
Receptionist	2 (4)
Psychologist	1 (2)
Self	2 (4)
Other	6 (13)
